# Parallel single B cell transcriptomics to elucidate pig B cell repertoire

**DOI:** 10.1038/s41598-024-65263-2

**Published:** 2024-07-10

**Authors:** Stanley Bram, Graeme Lindsey, Jenny Drnevich, Fangxiu Xu, Marcin Wozniak, Gisselle N. Medina, Angad P. Mehta

**Affiliations:** 1 Department of Chemistry, University of Illinois at Urbana-Champaign, 600 S Mathews Avenue, Urbana, IL 61801 USA; 2 Carl R. Woese Institute for Genomic Biology, University of Illinois at Urbana-Champaign, Urbana, IL USA; 3 Roy J. Carver Biotechnology Center, University of Illinois at Urbana-Champaign, Urbana, IL USA; 4 Cytometry and Microscopy to Omics Facility Roy J. Carver Biotechnology Center, University of Illinois at Urbana-Champaign, Urbana, IL USA; 5 National Agro and Bio-Defense Facility (NBAF), USDA, Manhattan, KS USA; 6 Plum Island Animal Disease Center, USDA, Orient Point, NY USA; 7 Cancer Center at Illinois, University of Illinois at Urbana-Champaign, Urbana, IL USA

**Keywords:** Biological techniques, Immunology, Molecular biology, Systems biology

## Abstract

Pork is the most widely consumed meat on the planet, placing swine health as a critical factor for both the world economy and the food industry. Infectious diseases in pigs not only threaten these sectors but also raise zoonotic concerns, as pigs can act as “mixing vessels” for several animals and human viruses and can lead to the emergence of new viruses that are capable of infecting humans. Several efforts are ongoing to develop pig vaccines, albeit with limited success. This has been largely attributed to the complex nature of pig infections and incomplete understanding of the pig immune responses. Additionally, pig has been suggested to be a good experimental model to study viral infections (e.g., human influenza). Despite the significant importance of studying pig immunology for developing infection models, zoonosis, and the crucial need to develop better swine vaccines, there is still very limited information on the response of the swine adaptive immune system to several emerging pathogens. Particularly, very little is known about the pig B cell repertoire upon infection. Understanding the B cell repertoire is especially crucial towards designing better vaccines, predicting zoonosis and can provide insights into developing new diagnostic agents. Here, we developed methods for performing parallel single pig B cell (up to 10,000 B cells) global and immunoglobulin transcriptome sequencing. We then adapted a computational pipeline previously built for human/mouse sequences, to now analyze pig sequences. This allowed us to comprehensively map the B cell repertoire and get paired antibody sequences from pigs in a single parallel sequencing experiment. We believe that these approaches will have significant implications for swine diseases, particularly in the context of swine mediated zoonosis and swine and human vaccine development.

## Introduction

More than one-third of the meat produced worldwide is pork, which makes it the most widely consumed meat on the planet^1,2^. Therefore, any infectious disease that affects pigs can threaten the economy of many countries. The high globalization of the swine industry amplifies this risk, facilitating the emergence and spread of different infectious agents including bacteria and viruses^3,4^. Several infectious viruses like influenza virus (IAV), Classical swine fever virus (CSFV), African swine fever virus (ASFV), Porcine reproductive and respiratory virus (PRRSV), Foot and mouth disease virus (FMD virus) and Pseudorabies virus (PRV), and infectious bacteria like *Brucella suis* pose a significant threat to global meat industry^5–7^. Several efforts are ongoing to develop countermeasures to combat these pathogens^8–10^. For example, development of subunit and live attenuated vaccines is one of the top priorities to combat ASFV, which poses a major threat to the global swine industry. Though several vaccine candidates have been evaluated, their efficacy remains variable, casting uncertainty on the future direction of vaccine development for swine diseases like ASF. This inconsistency has been largely attributed to the complex nature of ASFV infection and incomplete understanding of the host immune response. This highlights the need to study the host immune responses in pigs to viral infections^9^. Additionally, pigs also play an important role in zoonosis. They serve as “mixing vessels” for several animal and human viruses, which can lead to the emergence of new viruses that are capable of infecting humans. This phenomenon is particularly highlighted in the case of influenza where several strains of both human and avian influenza can infect swine, undergo reassortment within their hosts and lead to the emergence of new viruses with pandemic potential^11,12^ For example, in 2009 a new influenza strain jumped from pigs to humans resulting in a global pandemic^13,14^. Pigs have also been suggested to be a valuable experimental model to study human influenza and immune responses^15–18^. This is highlighted by observations like the same subtypes of influenza are endemic in both species, repeated exchanges of viruses between these hosts (swine to human and human to swine) and similarities in the clinical manifestation and pathogenesis. Furthermore, the distribution of influenza A virus receptors in the respiratory tract is similar in both pigs and humans^15^. Considering all of this, it has become imperative to study immune responses to zoonotic viruses in their zoonotic hosts (e.g., pig immune responses to zoonotic strains of influenza)^19^.

Despite the importance of pig immunology for model studies and the crucial need to develop better swine vaccines, there is still very limited information on the response of the pig adaptive immune system to several emerging pathogens. Particularly, very little is known about the pig B cell repertoire upon infection^20,21^. A comprehensive understanding of the B cell repertoire is especially crucial towards the rational design of improved vaccines and also can provide insights into developing new diagnostic tools^22^. To date, single-cell sequencing methods have been previously adapted to perform single B cell RNA sequencing in humans and mice^23–27^. However, these methods typically do not translate to other important animal species, mainly due to differences in the viability and distribution of B cells, diversity of B cell markers, diversity of immunoglobulin genes and lack of large data analysis methods available to analyze the diversity of immunoglobulin transcripts from various animals. This gap in methodological adaptation has significantly limited our ability to understand pig adaptive immune responses to infections (like ASFV and IAV) and vaccinations, thereby further limiting the development efficacious swine vaccines.

**Figure 1:**
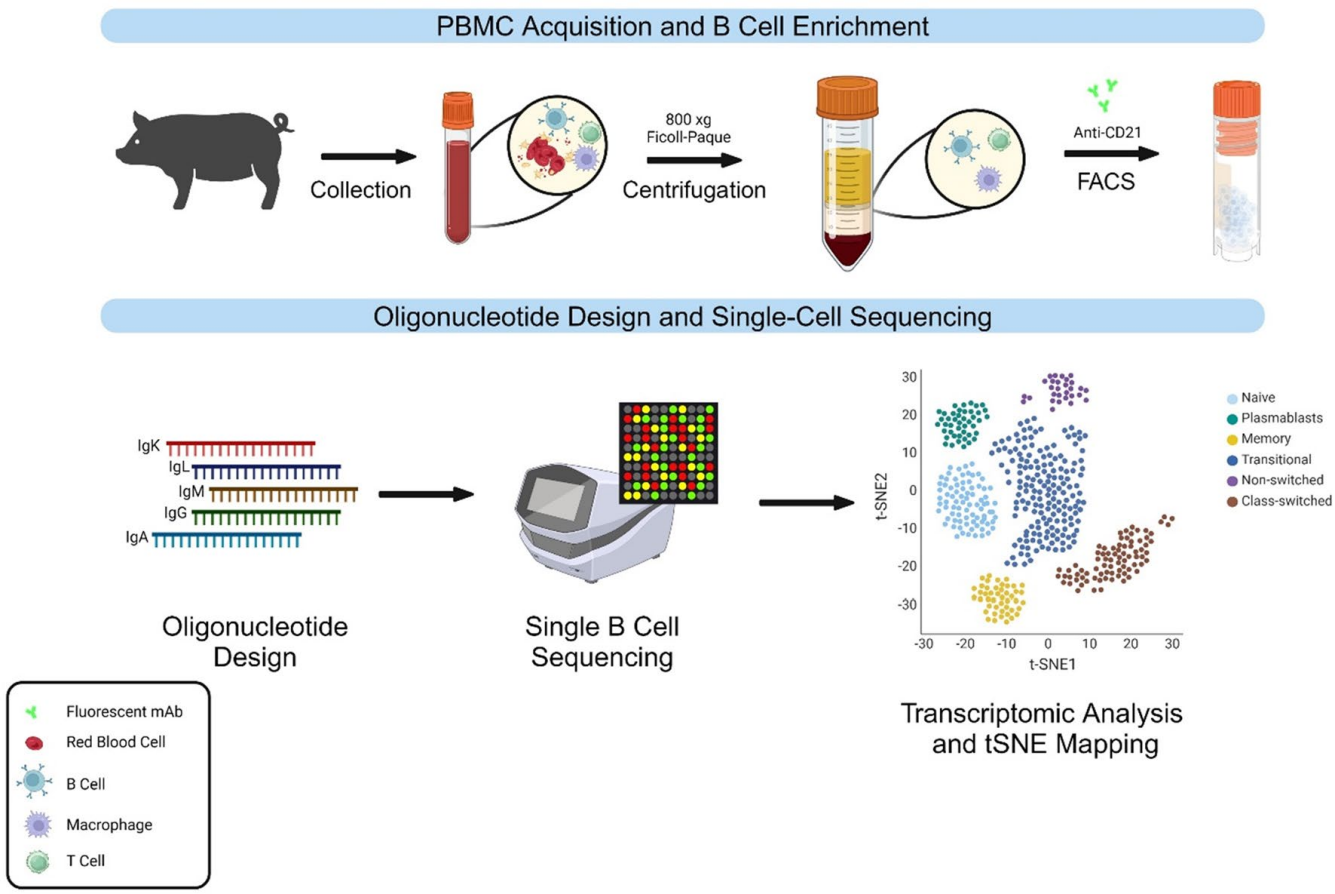
Workflow of the approach. The workflow of this approach spans initial blood collection from a swine specimen to separation of PBMC, sorting the B cells from the PBMC mixture, designing the correct oligonucleotides to amplify various immunoglobulin transcripts, sequencing of the single B cell transcripts using the ×10 Chromium platform, and concludes with transcriptomic analysis using the tSNE algorithm and bioinformatics analysis.

Here, we develop methods for parallel single cell sequencing of global and immunoglobulin-specific transcriptomes from up to 10,000 single pig B cells. The single B cell sequencing data was analyzed by adapting 10× Genomics' Cell Ranger pipelines to elucidate the pig B cell repertoire and obtain paired antibody sequences (Fig. 1). We first developed a workflow to prepare samples and reagents for sequencing. This involved identifying oligonucleotide sequences for immunoglobulin transcripts, isolating and preserving Peripheral Blood Mononuclear Cells (PBMC) from pig blood, using B cell markers to enrich live pig B cells, and adapting oligonucleotide sequences for the 10× Genomics 5' Chromium platform. We then performed single-cell sequencing to sequence immunoglobulin transcripts and global gene expression profile. We adapted the Cell Ranger pipeline to map V(D) J clonotypes and assess B cell diversity. Further, we performed bioinformatics studies to compare the enriched antibody sequences in our samples and detected the presence of antibody sequences that likely correspond to the PRRSV matured antibodies. These methods enable comprehensive mapping and analysis of the pig B cell repertoire in a single experiment. We believe that these methods will have a significant impact on studying swine disease, zoonosis, and vaccine development against swine and human viruses.

## Results

### Developing methods to amplify pig immunoglobulin transcripts from pig PBMC.

**Figure 2:**
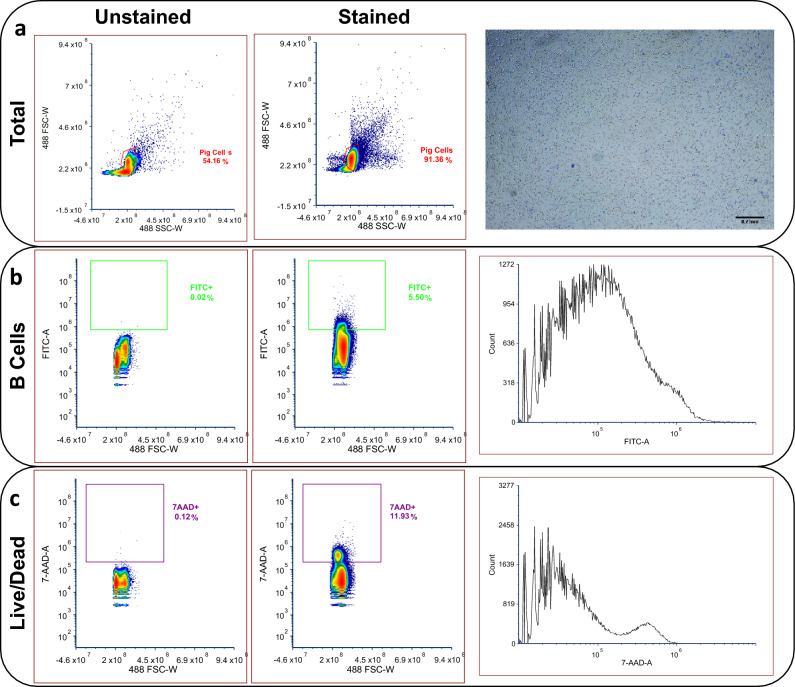
FACS sorting of B cells from the PBMC mixture. (**a**) PBMC were separated from peripheral blood by density centrifugation using Ficoll-Paque solution and imaged. (**b**) FITC-conjugated anti-CD21 mAb was used to sort B cells from the pool of PBMCs, with FACS data represented in histogram format. (**c**) 7-AAD was used as a dead-cell stain to apply extra stringency to the collected population of B cells, with FACS data represented in histogram format.

Our first goal was to identify a set of oligonucleotide sequences that would allow us to amplify and detect pig immunoglobulin transcripts. We began by collecting blood samples from *Sus scrofa* (domestic pigs) at the University of Illinois at Urbana-Champaign Meat Science Facility. The blood samples were collected in anticoagulant solution and were promptly chilled on ice or stored at refrigeration temperatures (2–8 °C) for a period of no more than 24–48 h. This was followed by isolation of PBMC by using a density gradient centrifugation technique with Ficoll-Paque media^28^. Post-centrifugation, the PBMC fraction, which was at the interphase between the plasma and Ficoll-Paque layers, was harvested. To further enrich and maintain the viability of the isolated PBMC, the plasma fraction was filtered through a 0.45 µm pore size filter to remove debris and was used as the wash medium following centrifugation steps. This plasma-based washing protocol was integral to preserving cellular viability, as our observations indicated a significant viability reduction (approximately 40%) when RPMI 1640 medium was used as an alternative. After the washing and viability assessments (Fig. 2a–c), surplus PBMCs were resuspended in autologous plasma supplemented with 5% (v/v) Dimethyl Sulfoxide (DMSO), followed by cryopreservation under controlled freezing conditions.

**Figure 3:**
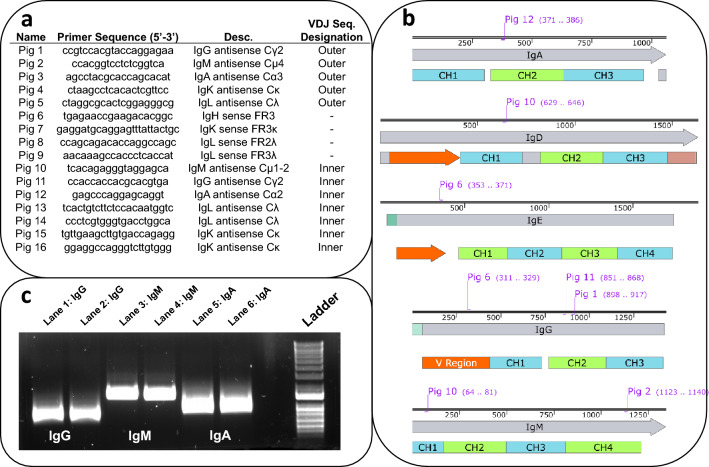
Oligonucleotide sequence selection and Ig sequence amplification. (**a**) Oligonucleotides were chosen based on their ability to hybridize with *Sus scrofa* Ig transcripts. (**b**) Sense oligonucleotides that recognized the FR regions were paired with antisense oligonucleotides that recognized constant regions of different Ig heavy and light subtypes. (**c**) Successful amplification of Ig heavy chain subtypes (IgG, IgM, IgA) yielded BLAST analyses that correlated with reported *Sus scrofa* Ig sequences (also see Supplementary Figs. 1, 3, 4).

Next, we identified and optimized a set of oligonucleotide sequences (Fig. 3a) to amplify and detect pig immunoglobulin transcripts (Fig. 3b). The selection of oligonucleotide sequences for the amplification of distinct antibody subtypes in this study was guided by methodologies established by Butler et al.^29^ in work that provided a comprehensive framework for the amplification of immunoglobulin heavy chain constant regions, specifically Cμ, Cα, and Cγ. In conjunction, oligonucleotide sequences to amplify light chains were designed referencing the published sequence of the *Sus scrofa* light chain^30^. Next, we used first-strand complementary DNA (cDNA) synthesis as a precursor to PCR amplification using the Superscript III First Strand Synthesis Kit adhering to the manufacturer's protocols. This was followed by the utilization of isotype-specific antisense oligonucleotide sequences in conjunction with a framework region 2 or framework 3 (FR2, FR3) oligonucleotide sequence located at the 5′ end for PCR. This oligonucleotide sequence combination was specifically designed to target and amplify the segments of the immunoglobulin heavy and/or light chain variable regions, facilitating the analysis of diverse antibody subtypes within the B cell repertoire. The amplification protocol was optimized as described in the methods section; these optimizations were critical for enhancing both the specificity and the yields (Fig. 3c, Supplementary Fig. 3). To verify that we were indeed amplifying immunoglobulin transcripts, we used Sanger sequencing of the amplicons followed by National Center for Biotechnology Information's (NCBI) Basic Local Alignment Search Tool (BLAST), to compare the sequenced amplicons against existing antibody subtype sequences in the database (Supplementary Fig. 1). The NCBI BLAST analysis revealed significant alignments of three query sequences with immunoglobulin (Ig) genes, specifically IgA, IgG, and IgM. The first sequence (Query_2684959) encoding the PCR product of IgA aligned from positions 9 to 770, covering the CH1, CH2, and CH3 regions of several reported IgA genes found in *S. scrofa*. The second sequence (Query_3728547) of the PCR product encoding IgG spanned positions 12 to 510, aligning with the FR1, FR3, and CH1 regions of several reported *S. scrofa* IgG genes, including a partial read of an annotated V region. The third sequence (Query_167739) encoding the PCR product of IgM extends from positions 7 to 1125, encompassing the CH1, CH2, CH3, and CH4 regions of several reported IgM genes in *S. scrofa*. Taken together, these alignments confirm successful amplification of Ig-encoding sequences by RT-PCR and validate the use of these oligonucleotides for preparing V(D)J libraries.

### Enrichment of live pig B cells from pig blood samples

Because it is crucial to have live cell population for single cell transcript sequencing, we next developed methods to isolate live pig B cells starting from cryopreserved stocks of pig PBMC. Frozen stocks of PBMCs were thawed at 37 °C and resuspended by pipetting. To detect and enrich B cells from PBMC, we used fluorescent antibody mediated Fluorescence-Activated Cell Sorting (FACS). Particularly, we used fluorescein isothiocyanate (FITC)-conjugated anti-CD21 monoclonal antibody for specifically labeling pig B cells in the bulk population of PBMC^31^. These samples were then analyzed by flow cytometry clearly revealing the population of fluorescently labeled cells corresponding to those expressing CD21 and binding to the FITC-anti-CD21 monoclonal antibody. For the establishment of accurate sorting parameters, an unlabeled control sample was utilized to set the baseline fluorescence. Sorting gates were then configured to select a population of cells exhibiting FITC fluorescence intensities surpassing the control by at least one to one and a half order of magnitude, ensuring high specificity in B cell identification. Additionally, 7-aminoactinomycin D (7AAD) staining was used as a viability marker to exclude any non-viable cells. Only cells that demonstrated negative staining for 7AAD and positive staining for FITC were included in the sorted population, enhancing the population of live B cells (Fig. 2b,c). In a detailed analysis of CD21+ (FITC+) B cells and dead (7-AAD+) lymphocytes using FACS, two distinct data collections at different sample volumes were conducted. For an average of 17,000 ± 2500 events, 9.73 ± 0.59% of the cells were identified as 7-AAD+, and 5.77 ± 1.06% were FITC+. The mean fluorescence intensity (F.I.) for 7-AAD was recorded at 581,000 ± 48,200 arbitrary units (a.u.), while the mean F.I. for FITC + cells was 1,350,000 ± 219,500 a.u. In a larger sample of 194,000 ± 40 events, the percentage of 7-AAD + cells increased to 13 ± 0.8%, with 6.23 ± 0.46% being FITC+. Here, the mean F.I. for 7-AAD was slightly lower at 480,000 ± 5560 a.u., and the mean F.I. for FITC was 1,240,000 ± 20,800 a.u. Additionally, an unstained control sample revealed mean F.I. values of 29,300 a.u. for 7-AAD and 53,900 a.u. for FITC; the mean F.I. of all stained cells exceeded the control by at least one order of magnitude.

### Single B cell sequencing to generate sequences corresponding to pig B cell repertoire

Our next goal was to perform parallel single B cell sequencing to specifically sequence immunoglobulin transcripts (by making V(D)J libraries) as well as sequence single B cell gene expression profile (by making Gene expression (GEX) libraries). Our rationale behind sequencing both single cell GEX and V(D)J libraries was to obtain a comprehensive dataset that reveals the diversity of cell types, states, and molecular dynamics within the sampled population. The V(D)J library targets the unique genetic recombination events and point mutations that occur in the variable (V), diversity (D), and joining (J) regions of immunoglobulin genes, revealing the clonality, diversity, and composition of the immune repertoire and the GEX library is designed to analyze the transcriptome at a single-cell level, providing high-resolution insights into the cell types and the gene expression profiles of individual cells within a heterogeneous sample. Combining single-cell sequencing information from GEX and V(D)J libraries through unique barcoding would allow us to obtain a detailed survey of the immune landscape, as well as the characterization of the B cell populations giving rise to it. As a result, it would be possible to observe from which B cell subtypes particularly enriched sequences and chain isotypes derive from. To this end, we began by preparing single-cell 5′ cDNA and V(D)J libraries. Briefly, single-cell suspensions were stained with acridine orange/propidium iodide (AO/PI), counted and checked for quality, debris content, and viability using a florescence-based cell counter. Cell suspensions with high viability (~ 81%, Supplementary Fig. 2) were then washed with PBS buffer containing 1.0% BSA, followed by removal of dead cells using Dead Cell Removal MicroBeads (Miltenyi Dead Cell Removal Kit). This method allows us to remove dead cells and early apoptotic cells with intact membranes, thereby facilitating the generation of ~ 85% live cell population (Supplementary Fig. 2).

The target number of cells (10,000) from each population were then converted into individually barcoded cDNA libraries with the Chromium NextGEM Single-Cell 5′ v2 kit from 10× Genomics (Pleasanton, CA) following the manufacturer’s protocols with the substitution of custom oligonucleotides for the amplification of pig V(D)J sequences (Pig17, Supplementary Table 1). Following double stranded-cDNA synthesis, we constructed individually barcoded dual-index libraries compatible with the Illumina chemistry and separate V(D)J amplifications were performed (Pig 1–5, Pig 10–16). The final libraries were quantitated, and their average size was determined. Thereafter, the libraries were pooled and diluted to the appropriate concentration for further quantitation by qPCR. The final 10 × single-cell gene expression and V(D)J library pool was sequenced on the Illumina NovaSeq X Plus system using one lane on the 10B flowcell as paired-reads with 150nt in length for a minimum of 100 k reads per cell for the gene expression and 10 k reads per cell for the V(D)J libraries. The first read of the single cell libraries is used for the UMI and 10 × barcode only, the 2nd read contains the RNA or V(D)J sequencing information. Basecalling and demultiplexing of raw data was done with the Illumina bcl2fastq v2.20 pipeline. Sorted data was further analyzed as described below.

### Adapting data analysis methods and using machine learning algorithms to determine the sequence distribution of pig immunoglobulin repertoire

To analyze both the V(D)J and gene expression (GEX) data, we used 10× Genomics’ Cell Ranger software (v7.2.0). Custom references corresponding to *Sus scrofa* genome (Sscrofa11.1) were made following 10×’s recommendations for the Gene Expression (GEX) data using Cell Ranger (mkref) on the Ensembl 110 Annotation. For the custom V(D)J reference was made using Cell Ranger (mkV(D)Jref) on Sscrofa11.1 and IMGT’s (the international ImMunoGeneTics database) IG and TR nucleotide database (v7.2.0). To jointly align the GEX and V(D)J data for cell calling and UMI quantification, Cell Ranger multi was run with the references and the 6 inner enrichment oligonucleotides. Results for the GEX and V(D)J sequences were further analyzed using 10× Genomics' Loupe V(D)J Browser (v5.1.0) as described below.

Chromium Single Cell Immune Profiling allows for more targeted investigations via custom gene expression panels that focus on specific genes of interest, and facilitates a granular analysis of the immunoglobulin repertoire at the single-cell level^32^. The analytical framework of this study encompassed the detailed examination of individual immunoglobulin heavy chain positive (IgH+) populations, with a specific focus on elucidating their associated light chain pairings. The goal of identifying and characterizing heavy chain sequences that exhibited significant enrichment within the sampled population, and subsequent mapping of their corresponding light chains, facilitated the reconstruction of the complete, physiologically relevant antibody sequences that result from the specimen, for example, existing in a disease state. The strength of this approach is marked by the cognate pairing of full-length nucleotide sequences corresponding to both heavy and light chains and the level of detailed sequencing that allowed for an in-depth examination of the entire antibody structure, encompassing the variable (V), diversity (D), and joining (J) segments. Such high-resolution analysis is crucial for understanding the intricacies of antigen recognition and antibody specificity, providing valuable insights into the molecular mechanics of the adaptive immune response^25^.

**Figure 4:**
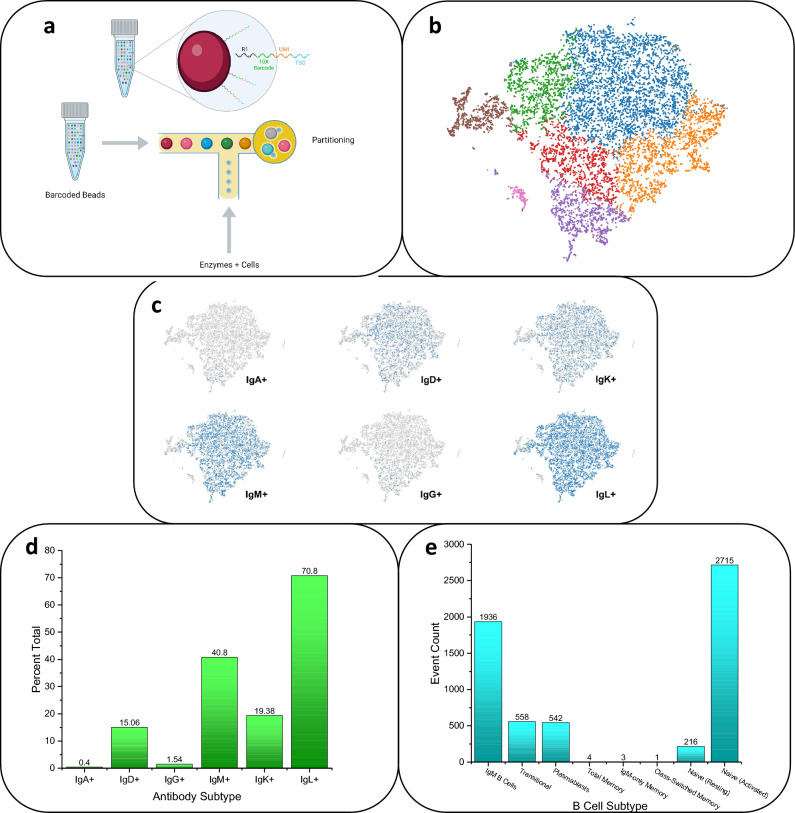
Workflow of single B cell sequencing. (**a**) The sequencing scheme passes DNA-barcoded beads through the microfluidic chip that incorporates cells and reverse transcription (RT) enzymes in a microemulsion drop; sufficient dilution of the cells across the microfluidic chip achieves single-cell resolution of V(D)J sequence pairs. (**b**) t-SNE clustering of cells based on their gene expression profiles and divided into 8 colored clusters using Cellranger's Graph-based method. (**c**) Categorization of the tSNE clustering by Ig-specific transcripts. (**d**) Graphical representation of visual clustering based on Ig-specific transcripts. (**e**) Graphical representation of tSNE clustering using multiple transcripts to deconvolute B populations in sorted B cell mixture.

The machine learning algorithm t-SNE, well-suited for the visualization of high-dimensional datasets, was employed to analyze the distribution of B cell subpopulations based on immunoglobulin isotype expression^33^. The resultant t-SNE plots provide a comprehensive visualization of the B cell repertoire, with each plot representing cells expressing a distinct Ig isotype. The t-SNE plots reveal distinct clusters of B cells positive for IgA (IgA+), IgD (IgD+), IgK (IgK+), IgM (IgM+), IgG (IgG+), and IgL (IgL+), as indicated by the blue markers. These markers stand in contrast to cells negative for these isotypes, which are depicted in gray. The heterogeneity within the B cell population is clearly observable, with each isotype defining a unique distribution pattern (Fig. 4a–e).

Quantitative analysis (Fig. 4b–e) indicates that within the sequenced sample, the IgL + cells are the most prevalent (70.8%), followed by IgM+ (40.8%), IgK+ (19.38%), IgD+ (15.06%), with a modest presence of IgG+ (1.54%), and an almost negligible amount of IgA + cells (0.4%). The high prevalence of IgL+ and IgK+ cells, representing the lambda (λ) and kappa (κ) light chains respectively, suggests significant diversity in antigen-binding specificity, which is indicative of a robust and adaptive immune response. The substantial number of IgM+ cells underscore their pivotal role in early immune responses and complement activation, serving as a crucial first line of defense against infections. The presence of IgD+ cells, though less abundant, highlights their involvement in the initiation and regulation of immune responses, particularly within the respiratory tract. Interestingly, the data reveal a lower proportion of IgG+ cells within the sequenced sample, which are essential for systemic immune functions such as opsonization, neutralization of toxins and viruses, and antibody-dependent cellular cytotoxicity (ADCC). The scant presence of IgA+ cells suggest a limited mucosal immune response in this sample, given IgA's critical role in protecting mucosal surfaces by neutralizing pathogens and preventing microbial adhesion, which can be reasoned as a tissue-specific immune response that would not be significantly detectable in a blood sample.

Overall, the data suggests that the B cell distribution is highly diversified, with each isotype contributing uniquely to the immune landscape. These patterns may reflect the functional specializations of B cells in pig immunity, with implications for both homeostatic immune regulation and pathological conditions.

### Evaluating the heterogeneity of B cell population

We also incorporated this methodology to map specific B cell populations, leveraging both phenotypic markers and transcriptomic profiles. CD19, a universally recognized B cell marker, plays a pivotal role in this mapping process. It is involved in forming the CD19/CD21 complex, a molecular assembly that synergistically enhances signal transduction through the B cell antigen receptor, particularly in response to T cell-dependent antigens that are tagged by the complement system. While CD21 was initially selected as our primary marker for B cell identification, its sequence data was not available in the reference database. Consequently, CD19 was utilized as an alternative marker due to its ubiquity in B cell transcriptomes. The classification of B cell subsets was conducted in accordance with the diagnostic standards established by the Mayo Clinic, which define B cell populations based on the expression of CD19 and other key surface markers or immunoglobulin isotypes.

**Figure 5:**
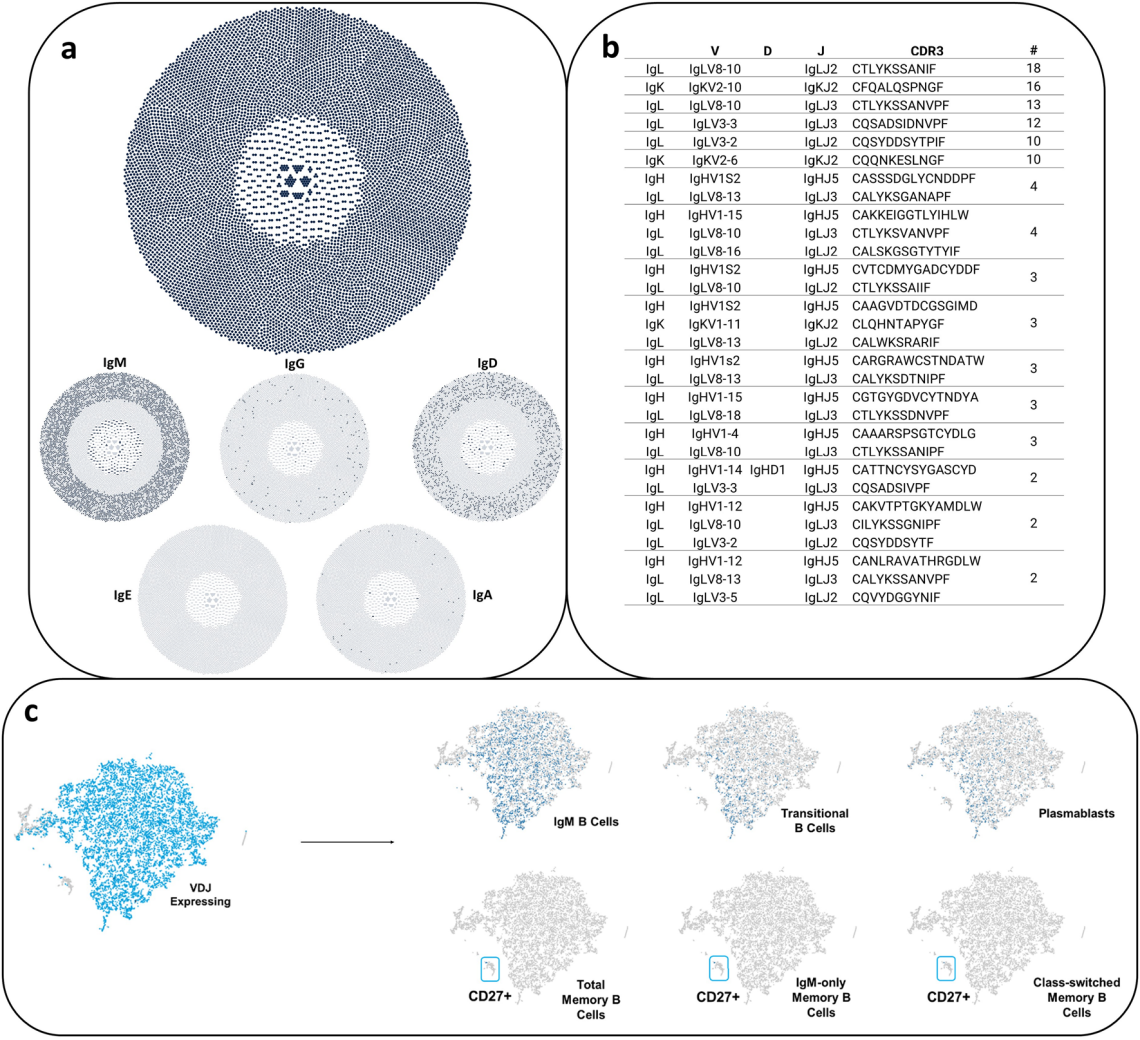
V(D)J Sequence Profiling and B Cell Mapping. (**a**) Generated cluster maps of paired sequences by Loupe VDJ Browser 5 and organization by IgH-specific subtypes within the pairings. (**b**) Typical table output displaying paired sequences, their corresponding CDRs post-SHM, their frequency of occurrence, and their germline sequence origin. (**c**) Categorization of the tSNE clustering by multiple transcripts to identify specific B cell populations.

The general B cell population was identified by CD19 expression (CD19+). B cells expressing IgM were classified as CD19+/IgM+, representing a fundamental subset of the B cell repertoire. Transitional B cells, which signify early stages of B cell development, were categorized as CD19+/IgM+/CD38+. Another critical subset, the plasmablasts, known for being antibody-secreting precursor cells, were delineated as CD19+/IgM−/CD38+. Further, total memory B cells, indicative of antigen-experienced B cells, were classified as CD19+/CD27+. Within the memory B cell compartment, distinct subsets were identified: IgM-only memory B cells, a unique group expressing solely IgM, were defined as CD19+/CD27+/IgM+/IgD−. Non-switched memory B cells, which have not undergone class-switch recombination, were identified as CD19+/CD27+/IgM+/IgD+. Finally, class-switched memory B cells, indicative of B cells that have undergone isotype switching to produce antibodies other than IgM, were categorized as CD19+/CD27+/IgM−/IgD− (see analysis in Fig. 5a–c).

The dense clustering of IgM B cells, as visualized in the t-SNE plot, suggests a homogenous population that can be primarily associated with two key stages in the B cell life cycle: naïve B cells and early responding B cells. Naïve B cells express membrane-bound IgM (and IgD) as their B cell receptors (BCR) and have yet to encounter their specific antigen. These cells are crucial for the immune system's ability to respond to a wide array of new infections. Upon encountering their specific antigen, these naïve B cells can become activated and either directly differentiate into IgM-secreting plasmablasts or enter the germinal center reaction to undergo class-switch recombination and somatic hypermutation. The homogeneity of this cluster could be indicative of the unselected nature of the naïve B cell repertoire, which encompasses a broad range of antigen specificities necessary for an effective primary immune response. The early responders, which are part of this IgM B cell subset, are critical as they provide the initial wave of antibody production during the adaptive immune response before more highly specialized and affinity-matured antibodies are produced.

Transitional B cells, represented by a more-diffuse cluster in the t-SNE plot, are typically recent emigrants from the bone marrow and are in the process of fully maturing into functional B cells within peripheral lymphoid organs, such as the spleen or lymph nodes. This clustering of transitional B cells suggests a discrete stage of development with a relatively uniform expression of surface markers that differ from both the bone marrow precursors and fully mature B cells. The density of the cluster may reflect the stringency of the selective processes that transitional B cells undergo; only those that successfully negotiate these checkpoints will survive and contribute to the mature B cell repertoire. The uniformity in their clustering pattern could also indicate a commonality in the expression of surface markers and other molecular features that define this particular developmental stage.

The dispersion of plasmablasts within the t-SNE plot reflects the dynamic and transitional nature of this B cell subset. Plasmablasts are effector cells that arise from activated B cells during an acute immune response. Their primary function is to produce and secrete large quantities of antibodies. This antibody production is crucial for controlling and clearing infections. While appearing diffuse, the population may be rationalized by representing a combination of their dynamic state of differentiation, the diversity of their B cell receptors due to somatic hypermutation, their migratory nature, and their role in producing antibodies during an ongoing immune response. This heterogeneity within the plasmablast population is essential, as it allows the immune system to rapidly produce a broad array of antibodies capable of neutralizing pathogens and facilitating an effective immune defense. This analysis highlights the complexity and distribution of swine B cell repertoire. Performing similar studies with infected pigs and vaccinated pigs could provide insights into swine B cell responses to pathogens and vaccines, swine mediated zoonosis and reverse zoonosis, and developing swine and human vaccines.

### Mapping V(D)J clonotypes

The t-SNE plots also reveal a heterogeneous landscape of B cell clonotypes. This diversity underscores the vast array of antigen specificities that the B cell population can recognize. Such resolution allows for the tracking of clonal lineages and the identification of dominant clonotypes, which may be critical in certain immune responses or diseases.

The sequence characteristics of each clonotype, detailing the V, D, and J gene segments, as well as the complementarity-determining regions (CDRs). The CDRs are particularly important as they directly interact with antigens, and their sequences are indicative of the clonotype's antigen-binding properties. Alignment also enables the identification of somatic hypermutations, a process vital for the affinity maturation of antibodies. We observed a rich diversity in the Ig gene segment usage and a high degree of somatic hypermutation, especially within the CDRs, suggesting an ongoing or past antigenic stimulus that has driven affinity maturation. While analyzing the sequenced immunoglobulin population, we observed multiple instances of recurrent immunoglobulin heavy chain and light chain pairing (Fig. 5a,b). This repetitive occurrence of identical IgH/IgL combinations across the sequenced cohort suggested a clonal expansion or selective enrichment of specific B-cell clones. Such findings indicate the presence of convergent immune responses, potentially driven by common antigenic stimuli. The detailed mapping of these clonotypes provides a framework for understanding how B cell diversity contributes to the immune response and may facilitate the identification of targets for vaccine design or antibody therapeutics. Therefore, future investigations into the dynamics of the B cell repertoire in health and disease may offer new avenues for immunological interventions.

### Analysis of the enriched antibody clonotypes

**Figure 6:**
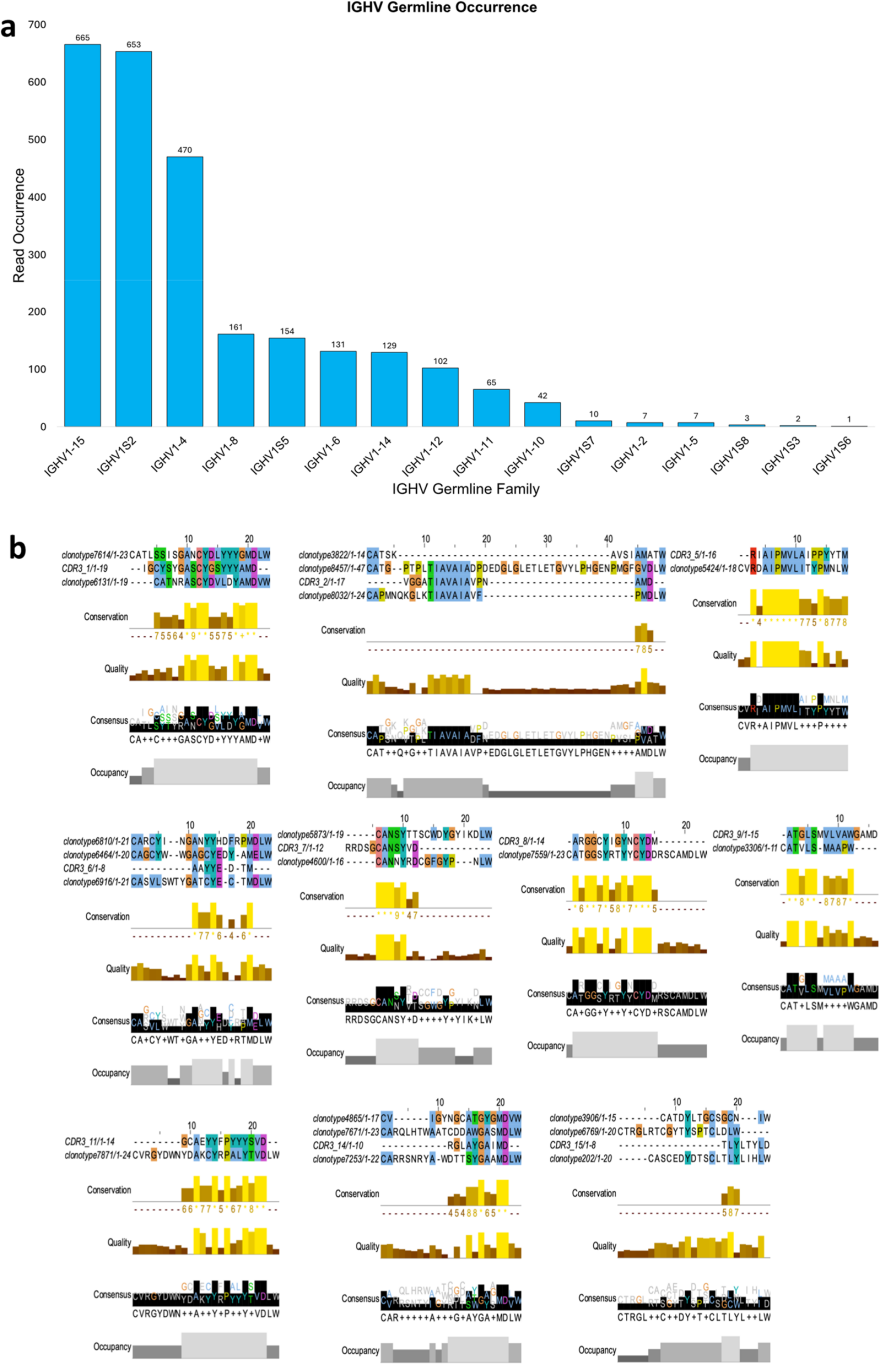

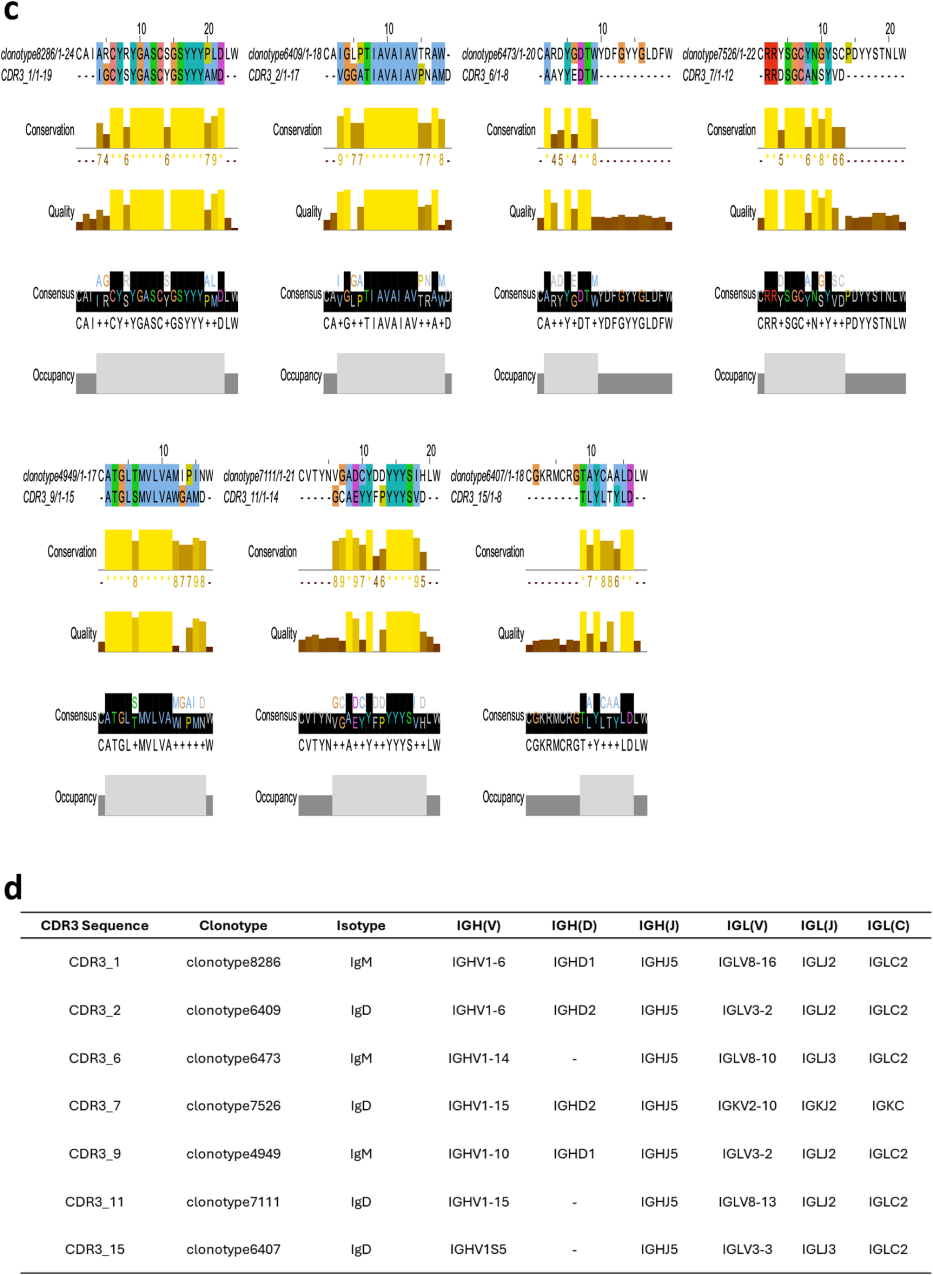
Analysis of V(D)J clonotypes: (**a**) Bar chart depicting the frequency of sequencing reads found for V genes of individual clonotypes as categorized by their germline sequence family. (**b**) Alignment of germline clonotypes that shared phylogenetic similarity with the enriched CDR3 sequences, when compared family to family. (**c**) Alignment of global clonotypes with the enriched CDR3 sequences. (**d**) Heavy/Light Chain pairings and corresponding V, D, J and isotype to each clonotype found by global phylogenetic analysis.

Next, we analyzed the observed antibody sequences to determine if there was any enrichment of antibody populations. To this end, we began by characterizing their germline origin believing the distribution of read occurrences for different IGHV germline families could highlight the enrichment of specific antibody sequences in a population. Derived from single-cell sequencing data, this analysis revealed that IGHV1-15, IGHV1S2, and IGHV1-4 were the most prominently enriched germline families, with read occurrences of 665, 653, and 470, respectively. These families significantly outnumbered other germline families such as IGHV1-8 and IGHV1-5, which had read occurrences of 161 and 154, respectively (Fig. 6a). The notable enrichment of these specific germline families indicated their predominant role in the immune repertoire of the pig specimen, potentially reflecting a higher frequency of antibodies derived from these families in response to antigen exposure.

We performed BLAST analysis on several CDR3 sequences from the most enriched germline family, IGHV1-15, and identified partial similarity to the CDR3 sequences with a previously reported study^34^ investigating the antibody response to in vivo challenge with a chimeric PRRSV antigen (Fig. 6b,c, see Supplementary Data files 1–4 for detailed sequence information). Given these interesting observations, we investigated if we could identify examples of convergent antibody evolution from two completely unique starting points, i.e., (i) domestic farm pigs from which we obtained pig blood samples, and (ii) previously reported analysis to find enriched antibody sequences in vivo challenge with a chimeric PRRSV antigen. To this end, a series of phylogenetic tree were constructed using a library of CDR3 sequences derived from each individual enriched germline family (IGHV1-15, IGHV1S2, IGHV1-4) (Supplementary Tables 2–4); within these trees was included a curated list of the enriched CDR3 sequences that emerged following exposure to the chimeric PRRSV antigen (Supplementary Fig. 5a–c). The analysis of the phylogenetic tree compared the 15 reported CDR3 sequences to assess their relative similarity to clonotypes within the enriched germline families. Of the 15 reportedly enriched CDR3 sequences, 10 exhibited direct phylogenetic similarity (sister groups) with clonotype variants derived from, at least, one of the three most abundantly enriched germline families (Supplementary Fig. 6). Distinct CDR3-germline alignments were identified for clonotypes originating from the IGHV1S2 family, specifically clonotypes 3306 and 7871, which corresponded to CDR3 sequences 9 and 11, respectively. Additionally, a unique clonotype from the IGHV1-15 family aligned with CDR3 sequence 5, and a single clonotype from the IGHV1-4 family aligned with CDR3 sequence 8. Further, several CDR3 sequences showed strong phylogenetic similarity with two of the three enriched germline families, specifically involving clonotypes from both the IGHV1-15 and IGHV1S2 families. Most notably, certain CDR3 sequences (CDR3: 2, 6, 14, 15) exhibited a direct phylogenetic relationship with clonotypes from each of the three highly enriched germline families (Fig. 6b). These sequences may indicate a conserved region within the CDR3 that is essential for the corresponding antibody response.

Next, we performed phylogenetic analysis to compare the CDR3 sequences across all germline families (Fig. 6c). This approach would offer more detailed insights into the conserved and variable regions within the enriched CDR3 sequences. By performing this analysis, we discovered that 7 out of the 15 germline sequences exhibited direct phylogenetic similarity with the provided CDR3 sequences (Supplementary Fig. 6, Supplementary Table 5). The extended regions of homology instill confidence that our findings correspond with previously reported^34^ enriched CDR3 sequences identified after PRRSV antigen challenge, likely indicating a convergence of antibody evolution. This suggests that it is possible that the individual from which we obtained the blood samples might have been infected with PRRSV at some point in its life, thereby resulting in the presence of CDR3 sequence motifs akin to those detected following antigen challenge in a different specimen. If this hypothesis is true, it could suggest a potential cross-reactivity and conservation of immune responses across different pigs.

Next, we used the Cell Ranger pipeline to obtain paired sequences of heavy and light chains, as well as the identification of heavy chain isotypes in the sequences that were identified above (Fig. 6d). Our analysis revealed that 3 of the 7 clonotypes originated from IgM chains, while the remaining 4 were derived from IgD chains. While IgM and IgD are co-expressed on the surface of non-activated B cells as antigen receptors, IgD is uniquely found in B cells that have begun migration from the bone marrow to germinal centers in lymphatic tissue. This observation supports the hypothesis that the observed clonotype may have arisen from a previous infection, possibly by PRRSV. The variable (V) region origins of the obtained clonotypes predominantly varied between the IGHV1-6 and IGHV1-15 families, with additional contributions from the IGHV1-14, IGHV1-10, and IGHV1S5 families. Interestingly, these clonotypes consistently selected the IGHJ5 family as the source for their joining (J) genes, with the interface between the J gene and the V gene forming the CDR3 region. The paired light chain sequences were mostly lambda chains, with 6 out of 7 clonotypes' V genes originating from either the IGLV3 or IGLV8 families. The J genes were either IGLJ2 or IGLJ3, and all constant (C) genes were from the IGLC2 family. The single kappa chain's V gene originated from the IGKV2-10 family, its J gene from the IGKJ2 family, and its C gene was IGKC (Fig. 6d).

## Discussion

Historically, single-cell sequencing has revealed significant insights into the genomics and the diversity of various cell populations, ranging from the microbiome to differentiated animal tissues. Furthermore, single-cell sequencing of RNA has facilitated the elucidation of cell-to-cell diversity within in a given population of cells. The enormous implications this technology has on human immunology, especially related to understanding the human B cell responses to infections, cannot be understated. Yet, while these platforms are well developed for human and mice samples, there are very limited resources to perform similar experiments for other relevant model animals or animals that play an important role in zoonosis and human diseases. This is particularly highlighted in the case of pig infections and zoonosis associated with pigs. As mentioned, pork is the most widely consumed meat on the plant and any infectious disease that affects pigs threaten the global economy and food industry. Additionally, swine play an important role in zoonosis as they can act as “mixing vessels” for several animal and human viruses and can lead to the emergence of new viruses that are capable of infecting humans. Vaccines are considered as one of the most important approaches to combat swine pathogens. Swine vaccines are currently being developed to combat ASFV which is one of the major threats to global swine industry. Despite significant efforts, the vaccine efficacy remains variable and the future direction of ASFV vaccine development remains unclear. Similarly, pig influenza vaccines are being developed to protect pigs from influenza and limit pig associated zoonosis. Additionally, pigs are considered as a good model system to study human influenza infections and develop and test human vaccines. Taken together, the importance of developing robust methods that would allow comprehensive elucidation of pig B cell repertoire upon infection must be emphasized.

In this study, we have furthered the methodologies for single B cell sequencing in pigs, integrating data analysis and machine learning approaches to elucidate the pig B cell repertoire. We first refined the methods for isolating and preserving PBMC from pig blood samples. Recognizing the importance of a live cell population for single-cell transcript sequencing, we used pig B cell makers to identify pig B cell population from PBMC and then developed cell sorting and collection methods to isolate live single pig B cells. We then generated barcoded oligonucleotide sequences to perform pig single B cells sequencing selectively sequence a variety of immunoglobulin transcripts. We adapted data analysis pipelines for analyzing swine sequences and used machine learning algorithms, which facilitated the acquisition of paired antibody sequences and map out V(D)J clonotypes, the diversity of antibody subtypes and the heterogeneity of B cell subtypes. Together these methods allowed us to comprehensively map the entire B cell repertoire of the enriched live B cell populations from pigs. We believe that performing similar studies with infected and vaccinated pigs could provide additional insights, potentially guiding the development of more efficacious vaccines. Further, our analysis has uncovered the differential clustering of B cell subpopulations highlighting the diverse phenotypes and specialized functions within the B cell repertoire. For example, comparative studies have been performed between human and mice B cell repertoires^35^. Such studies have revealed significant disparities in the composition and dynamics of nonmemory B cell pools. Human CD19 + IgM + B cells, while encompassing distinct populations corresponding to transitional 1 (T1), transitional 2 (T2), follicular mature, and marginal zone subsets, paralleling those in mice, contained a smaller fraction of transitional B cells in the adult human nonmemory B cell pool compared to their murine counterparts, with a particularly notable variance in the prevalence of T1 cells in bone marrow—over twice as frequent in mice. Despite the reduced representation of transitional B cells in humans, the conversion rate of these cells to naive follicular mature cells is markedly higher—three to sixfold across various tissues—than observed in mice. In pig peripheral blood samples, we find that transitional B cells represent a significant proportion of the overall naïve B cell population, akin to mice. We anticipate that our established protocols can be similarly expanded to B cell populations from other tissue samples (e.g., spleen). Conducting such experiments could further shed insights into differences in the B cell repertoires among different animals. Finally, we also analyzed the observed antibody sequences to determine if there was any enrichment of antibody populations. Our analysis revealed several CDR3 sequences from the most enriched germline family, IGHV1-15, and identified CDR3 sequences that were similar to the antibody sequences that were reported to be enriched following in vivo challenge with a chimeric PRRSV antigen. These analyses suggested that the pig from which we obtained the blood samples might have been infected with PRRSV at some point in its life, as evidenced by the presence of CDR3 sequence motifs akin to those detected following antigen challenge in a different pig. These observations could have implications on cross-reactivity and conservation of immune responses against PRRSV.

We believe that these approaches can be readily expanded to characterize the antibody repertoire of memory B cells. It is important to highlight that the CD21 marker is not ubiquitously expressed in all CD27+ cells (mature B cells). For example, some memory B cells, especially those that have undergone class switching, may express low levels or no CD21 at all. Several memory B cells are CD27+ but CD21−. We anticipate that one can readily use our approach in combination with appropriate antibodies to specifically target memory B cells (e.g., use of anti-CD27+ antibody) or target B cells more globally (e.g., use of anti-CD19 antibody). The differential clustering of these subpopulations illustrates the diverse phenotypes and specialized functions within the B cell repertoire. Our analysis underscores the complexity of the immune landscape and the importance of specific B cell subsets in maintaining immunological balance and mounting effective responses to pathogens. Understanding the distribution and characteristics of these subsets can inform vaccine strategies and therapeutic approaches targeting specific B cell-mediated immune responses.

## Materials and methods

### Blood collection and preparation

Blood samples were procured from an individual *Sus scrofa* (domestic pig) that was slaughtered at the University of Illinois at Urbana-Champaign Meat Science Facility during designated processing times. The authors did not directly handle animals but rather procured blood samples from this facility. The Meat Science Facility at the University of Illinois at Urbana-Champaign did not record information related to age, gender and breed. However, general records collected by the Meat Science Facility suggests that this individual comes from a set of pigs that were 172 days old. Additionally, the individual comes from a farm where the pigs were vaccinated at day 7 of age with Rhinishield, Mycoplasma hyosynoviae autogenous vaccine, and Enterisol Ileitis vaccine. At day 21 of age the receive Rhinishield, Mycoplasma hyosynoviae autogenous vaccine, Ingelvac Circoflex. After day 21 no other vaccines were given. Prior to collection, a volume of 250 mL of anticoagulant solution, pre-warmed to approximate physiological temperature, containing 750 mg of sodium EDTA in 2× PBS was prepared for immediate blood stabilization^36^. A total of 250 mL of blood was aseptically drawn into this solution, to a total volume of 500 mL. Visual inspection of the samples was conducted to ensure quality; ideal samples were characterized by a bright red coloration, while samples exhibiting a deeper, more sanguine shade were considered less optimal. Following procurement, the samples were promptly chilled on ice or stored at refrigeration temperatures (2–8 °C) for a period of no more than 24–48 h to maintain cellular integrity and prevent degradation. All experimental protocols for handling blood samples were approved by a UIUC Division of Research safety. All of the methods were carried out in accordance with relevant guidelines and regulations and in accordance with ARRIVE guidelines.

### Isolation of PBMC

Isolation of PBMC was achieved utilizing a density gradient centrifugation technique with Ficoll-Paque media^28^. In a sterile environment, 3 mL of Ficoll-Paque was carefully deposited at the bottom of a 15 mL conical tube. This was followed by the precise layering of 4 mL of the previously collected blood on top of the Ficoll-Paque layer, ensuring minimal disturbance of the density interface. The prepared tubes were subjected to centrifugation at a force of 800 × g for 40 min at room temperature, with the brake function deactivated to prevent disturbance of the formed layers.

Post-centrifugation, the PBMC fraction, discernible at the interphase between the plasma and Ficoll-Paque layers, was harvested. To further purify and maintain the viability of the isolated PBMCs, the plasma fraction—subjected to filtration through a 0.45 µm pore size filter to remove debris—was employed as a washing medium (1 mL per wash cycle). This plasma-based washing protocol was integral to preserving cellular viability, as preliminary observations indicated a significant viability reduction (approximately 40%) when RPMI 1640 medium was used as an alternative.

After washing and viability assessment, surplus PBMC were prepared for long-term storage. This was accomplished by resuspending the cells in autologous plasma supplemented with 5% (v/v) Dimethyl Sulfoxide (DMSO), followed by cryopreservation under controlled freezing conditions.

### FACS sorting of B cells

B cell isolation from the previously isolated PBMC was conducted using Fluorescence-Activated Cell Sorting (FACS). This process involved the labeling of B cells with a fluorescein isothiocyanate (FITC)-conjugated anti-CD21 monoclonal antibody, specifically targeting CD21 as a B cell marker^31^. The FACS procedure was optimized to maintain a controlled flow rate, not exceeding 3000 events per second, to ensure precision in cell sorting and minimize mechanical stress to the cells. For the establishment of sorting parameters, an unlabeled control sample was utilized to set the baseline fluorescence. Sorting gates were then configured to select for a population exhibiting FITC fluorescence intensities at least one to one and a half orders of magnitude greater than the control, ensuring high specificity in B cell identification. Additionally, 7-aminoactinomycin D (7AAD) staining was employed as a viability marker to exclude non-viable cells. Only cells that demonstrated negative staining for 7AAD and positive staining for FITC were included in the sorted population, enhancing the purity and viability of the isolated B cells. The cells were then washed with PBS buffer containing 1.0% BSA and the washed cells were subjected to dead cell removal. Dead cell removal (DCR) was performed using Dead Cell Removal MicroBeads (Miltenyi Dead Cell Removal Kit), which label surface markers of apoptotic and dead cells with magnetic beads. This method, effective for removing dead cells and early apoptotic cells with intact membranes, was chosen for its ability to selectively isolate viable cells from a sample. Briefly, magnetically labeled cells were passed through a separation column, where dead cells were retained, allowing living cells to be collected absent dead cells.

### Oligonucleotide selection and amplification of immunoglobulin transcripts.

The selection of oligonucleotides for the amplification of distinct antibody subtypes in this study was guided by methodologies established by Butler et al.^29^ in work that provided a comprehensive framework for the amplification of immunoglobulin heavy chain constant regions, specifically Cμ, Cα, and Cγ. In conjunction, oligonucleotides to amplify light chains were designed referencing the published sequence of the *Sus scrofa* light chain^30^. Adhering to the protocols delineated in the referenced study, first-strand complementary DNA (cDNA) synthesis was conducted as a precursor to PCR amplification using the Superscript III First Strand Synthesis Kit adhering to the manufacturer's protocols. Briefly, cDNA synthesis was initiated using total RNA as the starting material, with oligo(dT) oligonucleotides employed to selectively prime the reverse transcription of mRNA. This was followed by the utilization of isotype-specific antisense oligonucleotide sequences (Pig 10–16) in conjunction with a framework region 2 or framework 3 (FR2, FR3) oligonucleotide sequence (Pig 6–9) located at the 5′ end for PCR. This oligonucleotide sequence combination was specifically designed to target and amplify the segments of the immunoglobulin heavy and/or light chain variable regions, facilitating the analysis of diverse antibody subtypes within the B cell repertoire. The amplification of antibody subtypes from cDNA was performed using PrimeSTAR^®^ Max DNA Polymerase, selected for its high-fidelity amplification. Adhering largely to the manufacturer's guidelines, the PCR protocol was specifically adapted to the unique requirements of immunoglobulin gene sequences. Modifications included conducting 34 PCR cycles, an adjustment aimed at optimizing product yield and specificity. The annealing temperature was set at 64 °C to ensure precise oligonucleotide-template binding. Additionally, the annealing phase was consistently maintained at 30 s per cycle. These protocol optimizations were critical for enhancing both specificity and yield (Supplementary Figs. 3, 4).

Post-amplification, the generated amplicons were sequenced and a subsequent bioinformatic analysis was performed using the National Center for Biotechnology Information's (NCBI) Basic Local Alignment Search Tool (BLAST), to compare the sequenced amplicons against existing antibody subtype sequences in the database and validate the sequence integrity and specificity.

The process of library preparation was in adherence to manufacturer protocols [User Manual: Chromium Next GEM Single Cell 5′ Reagent Kits v2 (Dual Index)], but briefly utilized specially designed oligonucleotides that were combined with cellular contents to initiate cDNA synthesis. Oligonucleotides composed of a sequence for Illumina R1 (read 1 sequencing oligonucleotide), a 16 nucleotide 10 × Barcode, a 10 nucleotide unique molecular identifier (UMI), and a 13 nucleotide template switch oligo (TSO) were combined with cell lysate containing the necessary components for reverse transcription (RT), including RT reagents and poly(dT) RT oligonucleotides. The purification of the 10 × Barcoded first-strand cDNA from these mixtures was achieved using silane magnetic beads, which helped remove residual biochemical reagents and oligonucleotides, and the full-length cDNA underwent PCR amplification using outer oligonucleotides (Pig 1–5) and inner oligonucleotides (Pig 10–16) to target the constant regions of Ig transcripts.

### Construction of 10 × genomics 5′ RNA single cell libraries

Single-cell 5′ cDNA + V(D)J libraries were prepared at the DNA Services laboratory of the Roy J. Carver Biotechnology Center at the University of Illinois at Urbana-Champaign. Briefly, Single-cell suspensions were delivered to the Core lab, counted and checked for quality, debris content, and viability using the Nexcelom K2 brightfield/dual florescence cell counter (Nexcelom Biosciences, Lawrence MA) with AO/PI staining. Qualified cell suspensions were then washed with PBS buffer containing 1.0% BSA and recounted for library preparation. The target number of cells(10 k) from each population were then converted into individually barcoded cDNA libraries with the Chromium NextGEM Single-Cell 5′ v2 kit from 10X Genomics (Pleasanton, CA) following the manufacturer’s protocols with the substitution of custom oligonucleotides for pig V(D)J amplification (Pig 1–5, Pig 10–16).

Following ds-cDNA synthesis, individually barcoded dual-index libraries compatible with the Illumina chemistry were constructed and separate V(D)J amplifications performed. The final libraries were quantitated on Qubit (Life Technologies, Grand Island, NY) and the average size determined on the AATI Fragment Analyzer (Agilent Technologies, Santa Clara, CA). Libraries were pooled and the final pool diluted to 5 nM final concentration. The 5 nM dilution was then further quantitated by qPCR on a BioRad CFX Connect Real-Time System (Bio-Rad Laboratories, Inc. CA).

### Sequencing

The final 10 × single-cell gene expression and V(D)J library pool was sequenced on the Illumina NovaSeq X Plus system using one lane on the 10B flowcell as paired-reads with 150nt in length for a minimum of 100 k reads per cell for the gene expression and 10 k reads per cell for the V(D)J libraries. The first read of the single cell libraries is used for the UMI and 10 × barcode only, the 2nd read contains the RNA or V(D)J sequencing information. Basecalling and demultiplexing of raw data was done with the Illumina bcl2fastq v2.20 pipeline. Sorted data was posted to a password-secured AWS site for download and downstream processing.

### Supplementary Information

Supplementary Information 1.

Supplementary Information 2.

Supplementary Information 3.

Supplementary Information 4.

Supplementary Information 5.

## Data Availability

The datasets generated and/or analysed during the current study are available in the NCBI GEO repository, accession number: GSE269787.
