# Supplementary material for: Parallel single B cell transcriptomics to elucidate pig B cell repertoire
Source: Sci Rep. 2024 Jul 10;14:15997. doi: 10.1038/s41598-024-65263-2 (PMC11237004; doi:10.1038/s41598-024-65263-2)
Supplement: Supplementary file 1 — Supplementary Information 1. [file 41598_2024_65263_MOESM1_ESM.docx]

**Parallel Single-B cell Transcriptomics to Elucidate Swine B-cell Repertoire**

Stanley Bram^1,2^, Graeme Lindsey^1^, Jenny Drnevich^3^, Fangxiu Xu^3^, Marcin Wozniak^3^, Gisselle N. Medina^4^, Angad P. Mehta*^1,2,5^

^1^Department of Chemistry, University of Illinois at Urbana-Champaign, 600 S Mathews Avenue, Urbana, Illinois 61801, United States. ^2^Carl R. Woese Institute for Genomic Biology, University of Illinois at Urbana-Champaign. ^3^Roy J. Carver Biotechnology Center, University of Illinois at Urbana-Champaign. ^4^Plum Island Animal Disease Center, USDA. ^5^Cancer Center at Illinois, University of Illinois at Urbana-Champaign.

*Corresponding author: [apm8@illinois.edu](mailto:apm8@illinois.edu)

**Supporting Information**


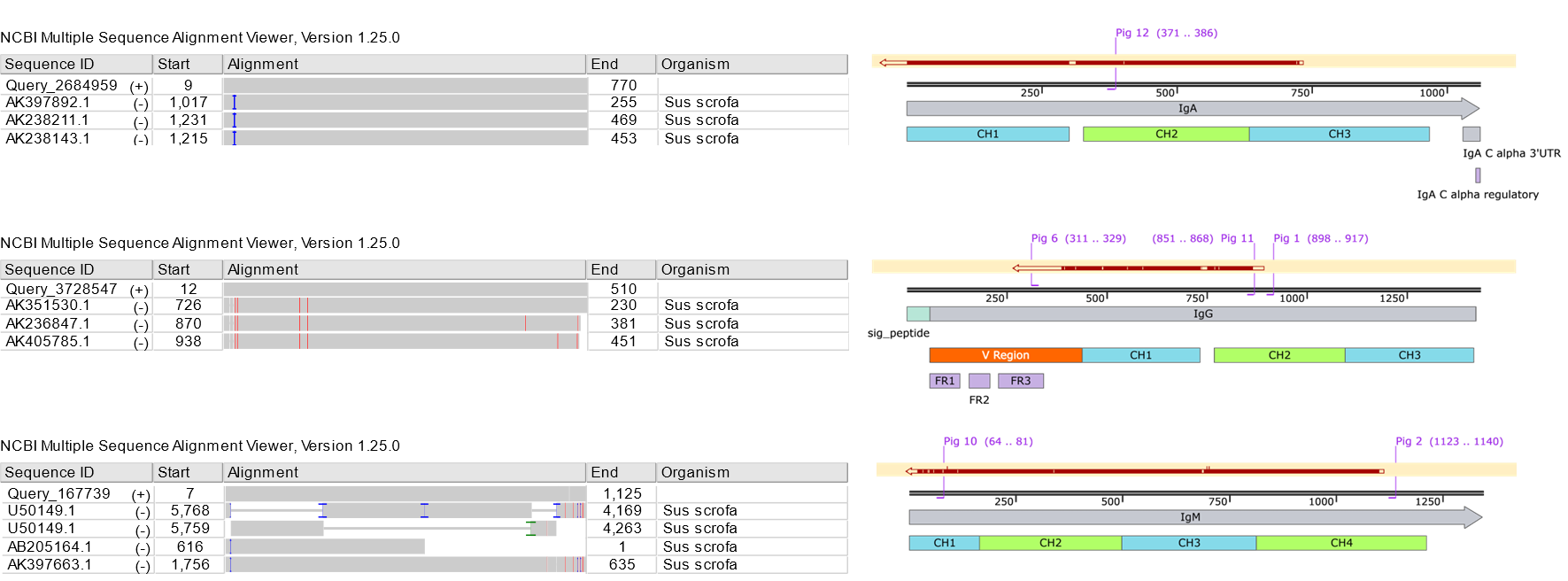


**Supplementary Figure 1: NCBI Blast Results**. Alignment of the PCR amplicons with sequenced transcripts in the NCBI GenBank yielded top results that correlated with immunoglobulin sequences for the heavy chain isotypes (IgA, IgG, IgM) from *S. scrofa*.

**Supplementary Figure 2**: **Live Cell Counts**. Total and Live cell counts during the different phases of single-cell sequencing sample preparation following FACS sorting.


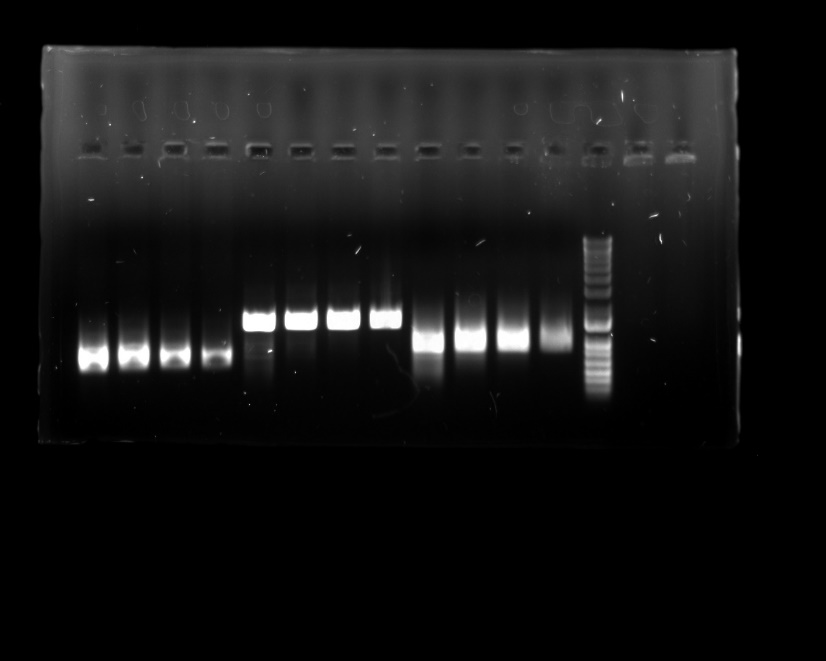


Ladder

Lane 11

Lane 10

Lane 5

Lane 6

Lane 7

Lane 8

Lane 9

Lane 4

Lane 3

Lane 2

Lane 1

Lane 12

**Supplementary Figure 3**: **Optimization of PCR**. Unedited gel image of oligonucleotide concentration optimization, by serial dilution, in the PCR to amplify IgG (Lane 1-4), IgM (Lane 5-8), and IgA (Lane 9-12); Oligonucleotides were serially diluted starting at 1.67 μM of each oligonucleotide.

**
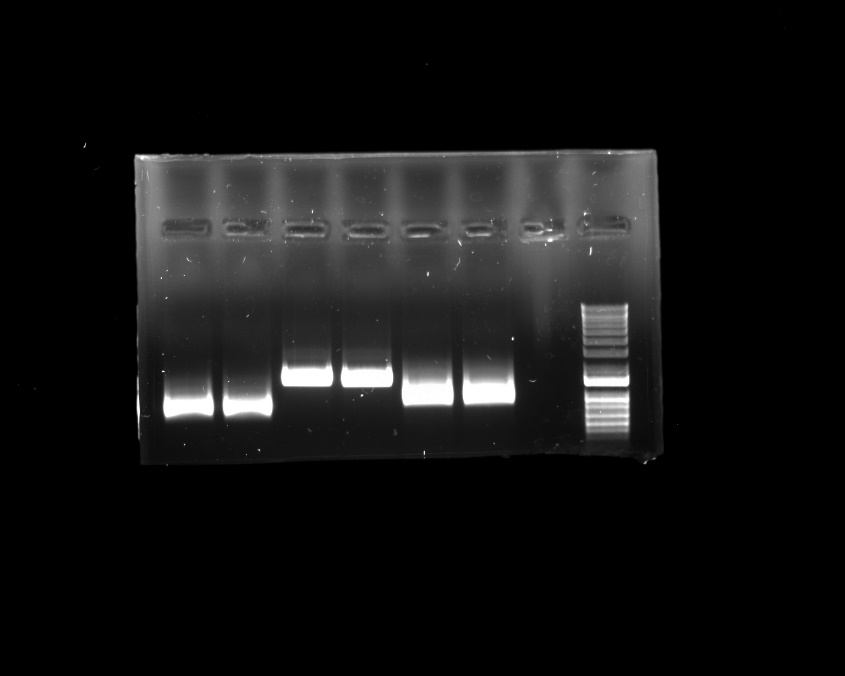
**

Ladder

Lane 6

Lane 4

Lane 5

Lane 3

Lane 2

Lane 1

**Supplementary Figure 4**: **Optimized PCR**. Unedited gel image of optimized PCR amplicons for IgG (Lane 1-2), IgM (Lane 3-4), and IgA (Lane 5-6).

**
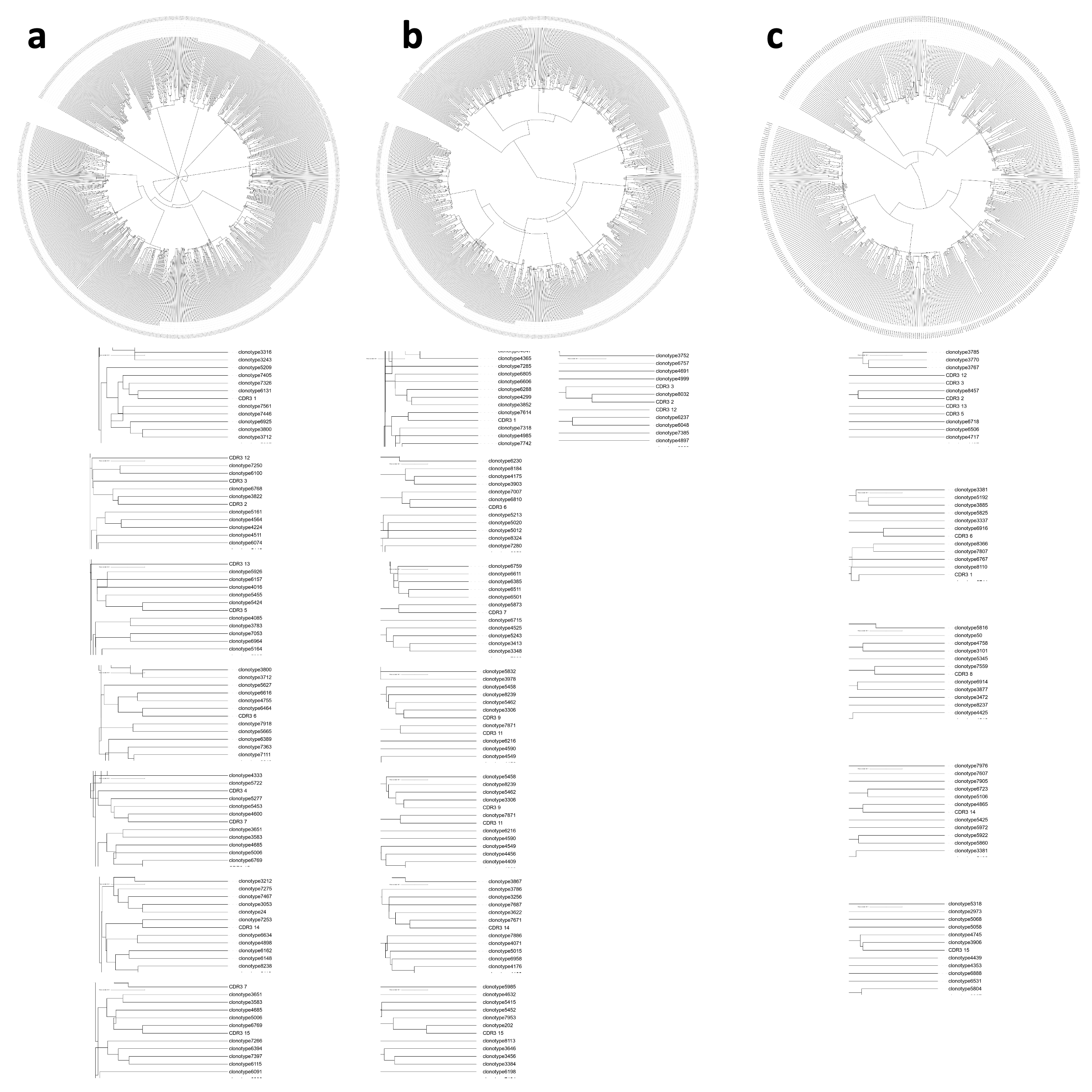
**

**Supplementary Figure 5**: **Phylogenetic tree analysis of enriched germline families**. Phylogenetic tree analysis for enriched CDR3 sequences in relation to a) IGHV1-15 family, b) IGHV1S2 family, and c) IGHV-1-4 family. Detailed sequences corresponding to each clonotype are in Supplementary data files 1-4.

**
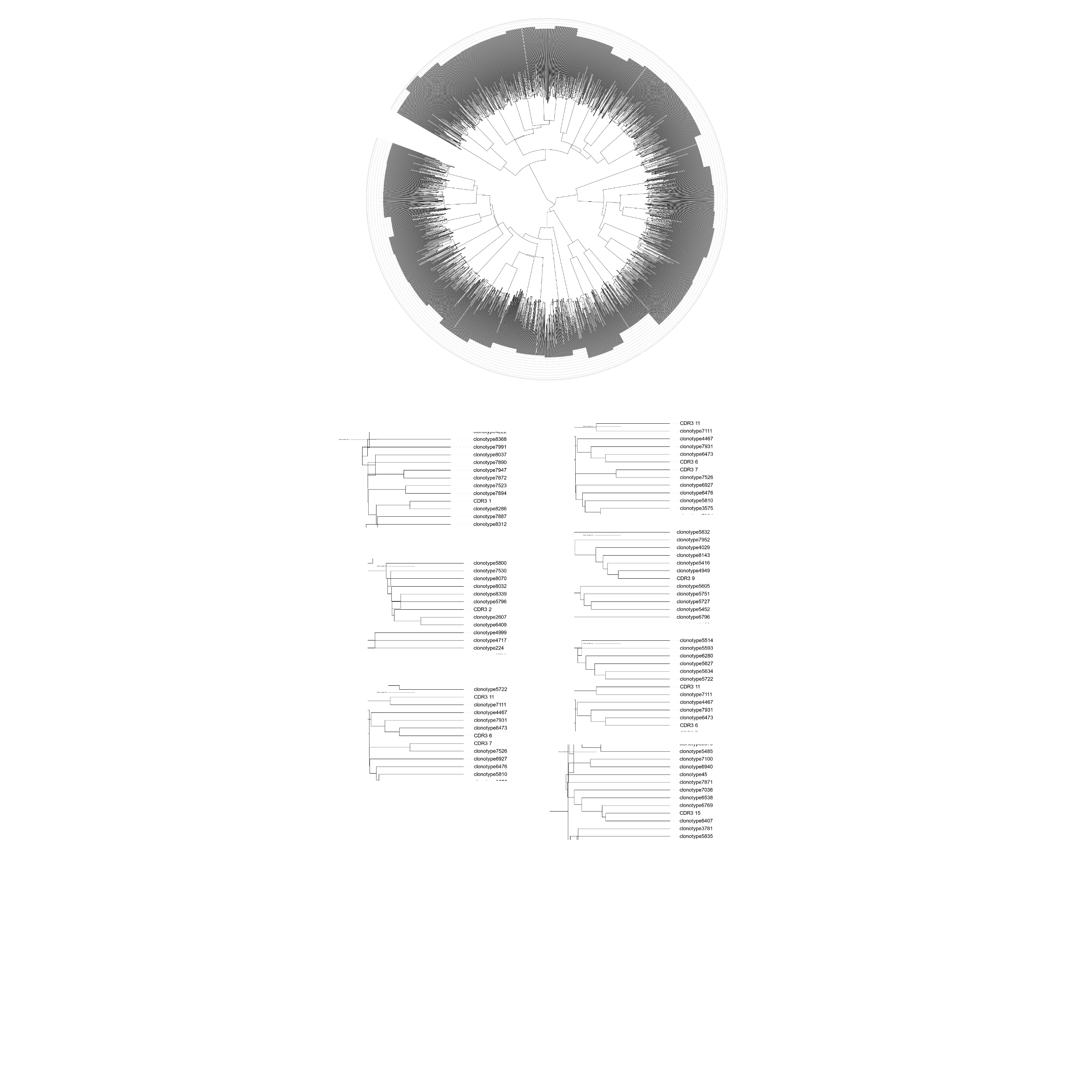
**

**Supplementary Figure 6: Phylogenetic tree analysis across all germline families.** Phylogenetic tree analysis for enriched CDR3 sequences in relation to the complete set of clonotypes. Detailed sequences corresponding to each clonotype are in Supplementary data files 1-4.

**Supplementary Table 1: Oligonucleotides used in this study.** Oligonucleotides specific for S. scrofa Ig transcripts were obtained and used according to their descriptions and VDJ sequencing designations for outer and inner oligonucleotide pools.

| *Name* | *Oligonucleotide Sequence (5’-3’)* | *Desc.* | *VDJ Seq. Designation* |
| --- | --- | --- | --- |
| *Pig 1* | ccgtccacgtaccaggagaa | IgG antisense Cγ2 | Outer |
| *Pig 2* | ccacggtcctctcggtca | IgM antisense Cμ4 | Outer |
| *Pig 3* | agcctacgcaccagcacat | IgA antisense Cα3 | Outer |
| *Pig 4* | ctaagcctcacactcgttcc | IgK antisense Cκ | Outer |
| *Pig 5* | ctaggcgcactcggagggcg | IgL antisense Cλ | Outer |
| *Pig 6* | tgagaaccgaagacacggc | IgH sense FR3 | - |
| *Pig 7* | gaggatgcaggagtttattactgc | IgK sense FR3κ | - |
| *Pig 8* | ccagcagacaccaggccagc | IgL sense FR2λ | - |
| *Pig 9* | aacaaagccaccctcaccat | IgL sense FR3λ | - |
| *Pig 10* | tcacagagggtaggagca | IgM antisense Cμ1-2 | Inner |
| *Pig 11* | ccaccaccacgcacgtga | IgG antisense Cγ2 | Inner |
| *Pig 12* | gagcccaggagcaggt | IgA antisense Cα2 | Inner |
| *Pig 13* | tcactgtcttctccacaatggtc | IgL antisense Cλ | Inner |
| *Pig 14* | ccctcgtgggtgacctggca | IgL antisense Cλ | Inner |
| *Pig 15* | tgttgaagcttgtgaccagagg | IgK antisense Cκ | Inner |
| *Pig 16* | ggaggccagggtcttgtggg | IgK antisense Cκ | Inner |
| *Pig 17* | gatctacactctttccctacacgacgc | 10X specific | Universal 10X 5’ oligo |

**Supplementary Table 2: IGHV1-15 branches**. Nearest neighbors and CDR3 sequences for enriched CDR3s in the IGHV1-15 family.

|  | **CDR3 Sequence** | **IGHV1-15** | **CDR3 Sequence** |
| --- | --- | --- | --- |
| **CDR3_1** | IGCYSYGASCYGSYYYAMD | clonotype6131 | CATNRASCYDVLDYAMDVW |
| **CDR3_2** | VGGATIAVAIAVPNAMD | clonotype3822 | CATSKAVSIAMATW |
| **CDR3_3** | SAVAIAVTFGGRQQYYAMD | n/a | n/a |
| **CDR3_4** | GCPLYSGCYIGQLGGVMD | n/a | n/a |
| **CDR3_5** | RIAIPMVLAIPPYYTM | clonotype5424 | CVRDAIPMVLITYPMNLW |
| **CDR3_6** | AAYYEDTM | clonotype6464 | CAGCYWWGAGCYEDYAMELW |
| **CDR3_7** | RRDSGCANSYVD | clonotype4600 | CANNYRDCGFGYPNLW |
| **CDR3_8** | ARGGCYIGYNCYDM | n/a | n/a |
| **CDR3_9** | ATGLSMVLVAWGAMD | n/a | n/a |
| **CDR3_10** | RDDFSDYCSASVCGME | n/a | n/a |
| **CDR3_11** | GCAEYYFPYYYSVD | n/a | n/a |
| **CDR3_12** | MSYTYGISYDYCGMDR | n/a | n/a |
| **CDR3_13** | TGLMVLSSRTYGAMD | n/a | n/a |
| **CDR3_14** | RGLAYGAIMD | clonotype7253 | CARRSNRYAWDTTSYGAAMDLW |
| **CDR3_15** | TLYLTYLD | clonotype6769 | CTRGLRTCGYTYSPTCLDLW |

**Supplementary Table 3: IGHV1S2 branches**. Nearest neighbors and CDR3 sequences for enriched CDR3s in the IGHV1S2 family.

|  | **CDR3 Sequence** | **IGHV1S2** | **CDR3 Sequence** |
| --- | --- | --- | --- |
| **CDR3_1** | IGCYSYGASCYGSYYYAMD | clonotype7614 | CATLSSISGANCYDLYYYGMDLW |
| **CDR3_2** | VGGATIAVAIAVPNAMD | clonotype8032 | CAPMNQKGLKTIAVAIAVFPMDLW |
| **CDR3_3** | SAVAIAVTFGGRQQYYAMD | n/a | n/a |
| **CDR3_4** | GCPLYSGCYIGQLGGVMD | n/a | n/a |
| **CDR3_5** | RIAIPMVLAIPPYYTM | n/a | n/a |
| **CDR3_6** | AAYYEDTM | clonotype6810 | CARCYINGANYYHDFRPMDLW |
| **CDR3_7** | RRDSGCANSYVD | clonotype5873 | CANSYTTSCWDYGYIKDLW |
| **CDR3_8** | ARGGCYIGYNCYDM | n/a | n/a |
| **CDR3_9** | ATGLSMVLVAWGAMD | clonotype3306 | CATVLSMAAPW |
| **CDR3_10** | RDDFSDYCSASVCGME | n/a | n/a |
| **CDR3_11** | GCAEYYFPYYYSVD | clonotype7871 | CVRGYDWNYDAKCYRPALYTVDLW |
| **CDR3_12** | MSYTYGISYDYCGMDR | n/a | n/a |
| **CDR3_13** | TGLMVLSSRTYGAMD | n/a | n/a |
| **CDR3_14** | RGLAYGAIMD | clonotype7671 | CARQLHTWAATCDDAWGASMDLW |
| **CDR3_15** | TLYLTYLD | clonotype202 | CASCEDYDTSCLTLYLIHLW |

**Supplementary Table 4: IGHV1-4 branches**. Nearest neighbors and CDR3 sequences for enriched CDR3s in the IGHV1-4 family.

|  | **CDR3 Sequence** | **IGHV1-4** | **CDR3 Sequence** |
| --- | --- | --- | --- |
| **CDR3_1** | IGCYSYGASCYGSYYYAMD | n/a | n/a |
| **CDR3_2** | VGGATIAVAIAVPNAMD | clonotype8457 | CATGPTPLTIAVAIADPDEDGLG LETLETGVYLPHGENPMGFGVDLW |
| **CDR3_3** | SAVAIAVTFGGRQQYYAMD | n/a | n/a |
| **CDR3_4** | GCPLYSGCYIGQLGGVMD | n/a | n/a |
| **CDR3_5** | RIAIPMVLAIPPYYTM | n/a | n/a |
| **CDR3_6** | AAYYEDTM | clonotype6916 | CASVLSWTYGATCYECTMDLW |
| **CDR3_7** | RRDSGCANSYVD | n/a | n/a |
| **CDR3_8** | ARGGCYIGYNCYDM | clonotype7559 | CATGGSYRTYYCYDDRSCAMDLW |
| **CDR3_9** | ATGLSMVLVAWGAMD | n/a | n/a |
| **CDR3_10** | RDDFSDYCSASVCGME | n/a | n/a |
| **CDR3_11** | GCAEYYFPYYYSVD | n/a | n/a |
| **CDR3_12** | MSYTYGISYDYCGMDR | n/a | n/a |
| **CDR3_13** | TGLMVLSSRTYGAMD | n/a | n/a |
| **CDR3_14** | RGLAYGAIMD | clonotype4865 | CVIGYNGCATGYGMDVW |
| **CDR3_15** | TLYLTYLD | clonotype3906 | CATDYLTGCSGCNIW |

**Supplementary Table 5: Global branches**. Nearest neighbors and CDR3 sequences for enriched CDR3s in the across all germline families.

|  | **CDR3 Sequence** | **ALL** | **CDR3 Sequence** |
| --- | --- | --- | --- |
| **CDR3_1** | IGCYSYGASCYGSYYYAMD | clonotype8286 | CAIARCYRYGASCSGSYYYPLDLW |
| **CDR3_2** | VGGATIAVAIAVPNAMD | clonotype6409 | CAIGLPTIAVAIAVTRAW |
| **CDR3_3** | SAVAIAVTFGGRQQYYAMD | n/a | n/a |
| **CDR3_4** | GCPLYSGCYIGQLGGVMD | n/a | n/a |
| **CDR3_5** | RIAIPMVLAIPPYYTM | n/a | n/a |
| **CDR3_6** | AAYYEDTM | clonotype6473 | CARDYGDTWYDFGYYGLDFW |
| **CDR3_7** | RRDSGCANSYVD | clonotype7526 | CRRYSGCYNGYSCPDYYSTNLW |
| **CDR3_8** | ARGGCYIGYNCYDM | n/a | n/a |
| **CDR3_9** | ATGLSMVLVAWGAMD | clonotype4949 | CATGLTMVLVAMIPINW |
| **CDR3_10** | RDDFSDYCSASVCGME | n/a | n/a |
| **CDR3_11** | GCAEYYFPYYYSVD | clonotype7111 | CVTYNVGADCYDDYYYSIHLW |
| **CDR3_12** | MSYTYGISYDYCGMDR | n/a | n/a |
| **CDR3_13** | TGLMVLSSRTYGAMD | n/a | n/a |
| **CDR3_14** | RGLAYGAIMD | n/a | n/a |
| **CDR3_15** | TLYLTYLD | clonotype6407 | CGKRMCRGTAYCAALDLW |
